# Attacks on medical workers in Syria: Implications for conflict research

**DOI:** 10.1371/journal.pmed.1002560

**Published:** 2018-04-24

**Authors:** Michael Spagat

**Affiliations:** Department of Economics, Royal Holloway, University of London, Egham, Surrey, United Kingdom

## Abstract

In a Perspective linked to the Research Article by Haar and colleagues, Michael Spagat discusses the challenges and importance of conducting research on mortality in regions affected by violent conflicts.

The ongoing war in Syria has raged for seven years with no end in sight. Through news media and social media, the rest of the world learns of horrific attacks on health workers and medical facilities, but a complete account of these events does not escape Syria. Journalists operating in Syria also face great dangers; consequently, journalistic coverage of the Syrian war is patchy, compromising our ability to collect event data that can underpin a robust research program on the human costs of the war (though there are some strong research studies, including [1–4]). Social media plugs some of the coverage gaps, but we lack validated tools for separating reliable social media posts from unreliable ones. Due to these limitations, research into the Syrian war often must rely on relatively random fragments of information of unknown quality.

In this week’s *PLOS Medicine*, Rohini Haar and colleagues [5] describe an extraordinary field-based data collection effort conducted amidst the ongoing war in Syria, spearheaded by the Syrian American Medical Society (SAMS) and supported by a team of mainly United States-based researchers. The resulting database records the deaths of 112 health workers and 185 patients that occurred as part of more than 200 attacks on healthcare facilities and transport vehicles in the governorates of Aleppo, Idlib, Hama, and Homs in 2016. The paper also matches the SAMS-documented events against those recorded in a separate but similar database maintained by Physicians for Human Rights (PHR) using open-source methods [6]. The SAMS–PHR overlap is substantial but not complete, suggesting that there are further events not captured by either data-gathering project.

## Attacks on health facilities in Syria

Haar and colleagues show that violent, often lethal, attacks on health infrastructure, workers, and patients were very common in northern Syria in 2016 [5]. Though the scope of this study was limited to four governorates, this violence almost certainly continues to the present throughout much of the country. Moreover, such attacks occurred more frequently than we might reasonably have believed based on the prior evidence that was available [6]. These carefully documented events represent serious and widespread violations of International Humanitarian Law (IHL) with tragic and morally reprehensible consequences. To be sure, the 297 deaths documented in the paper constitute a small percentage of total violence in Syria in 2016 (see, for example, the database of the Violations Documentation Center in Syria [7] and the analysis of [2]). Indeed, attacks in Syria frequently kill children [1,3,4], with the widespread use of explosives in densely populated areas posing a particular hazard [3,4]. Children, medical units, and personnel are all specially protected under IHL [8,9,10], so the violations documented in the paper and [1,3,4] have special salience. Moreover, these attacks on health infrastructure might have exacerbated effects on health by impeding people in a conflict-affected environment from seeking and providing medical care. Indeed, a striking observation of Haar and colleagues [5] is that only a low proportion of medical facilities and vehicles are labelled as such in Syria, rendering them hard to find both for potential patients and would-be attackers, perhaps intentionally in the latter case.

We also learn much from the study about the variety of channels available to capture news of violent events in Syria. Specifically, the comparison of events in the PHR system with events in the SAMS system is illuminating and could become more so with some further work. There are two issues that are important to bear in mind when making these comparisons. First, sources underpinning the two databases are very different: SAMS events are reported by a network of medical field workers, while PHR’s data come from researchers scouring a wide range of open sources on the internet. Second, the inclusion criteria for the two systems differ, so we must be careful not to make inappropriate comparisons and thus draw incorrect conclusions about the potential for capturing certain types of events through one source or another. For example, PHR covers the whole of Syria from March 22, 2011, through the end of 2017 and continues to be updated, whereas SAMS covers four governorates in 2016. PHR covers attacks only to medical facilities, whereas SAMS includes medical vehicles in addition to facilities.

The PHR database documents 492 attacks and 847 medical personnel killed throughout Syria until the end of 2017 [6]. Therefore, prior to Haar and colleagues’ work, we already knew that medical facilities and workers were under widespread attack. Yet the SAMS data bring several new aspects to the table. First, the field reporting system corroborates 60 out of a possible 90 comparable PHR-recorded incidents. This is reassuring given the potential for fake incidents to be reported online. PHR protects itself from spurious news by requiring three-source confirmation for its incidents, but it is nice to know that this confirmation system seems to be doing its job. Second, SAMS finds 117 comparable incidents not captured by PHR, suggesting that violence is more common than had been documented previously.

Each database has advantages over the other. As mentioned above, PHR has a broader timeframe and geographic scope than SAMS. SAMS seems more complete than PHR for events they both try to cover, although neither database is comprehensive. Cost comparisons are tricky, since SAMS piggybacks off of existing field presence. The cost of creating this presence from scratch must be huge, but once in place, it is relatively straightforward to put it to work collecting the data. Nevertheless, it seems that some of the SAMS field workers found the mobile data-entry instrument to be onerous and bypassed it for about half of their reports in Haar and colleagues’ study.

## What next?

The work of PHR, SAMS, and conflict data projects in general could benefit greatly from deeper research into event matching across the two databases. Moreover, Haar and colleagues mention in passing that there is a third data-collection system operated by the UN Health Cluster for northern Syria. A three-way matching comparison would be better still.

First, PHR uses a very demanding triple-source confirmation system, so researchers must have records of many real events that did not make it into their database due to insufficient corroboration. It would be useful to check these potential data points against the SAMS database for possible corroboration. More generally, PHR can assign reliability ratings to each of its open sources by cross-checking events reported by each source against the fieldwork-based events of SAMS. For example, one might view Twitter accounts as inherently questionable information sources since it is so easy to set one up and start tweeting. However, if the events reported by a Twitter account are consistently confirmed through field work, then it could make sense for PHR to take tweets from that account at face value without requiring event-by-event corroboration. Conversely, in-depth matching could discredit certain sources, removing the need to monitor them in the future. Second, SAMS should examine PHR-reported events not captured by SAMS as potentially offering clues to gaps in their field networks. Finally, validation of open-source information types through medical field work can have implications for documenting violence in the Syrian war more generally. If, for example, certain social media accounts are accurate about attacks on medical facilities, then we can infer that other information they provide is also likely to be accurate.

We all owe a great debt to the contributors to this article. I sincerely hope that the team continues its efforts and that the violence affecting their work abates. Meanwhile, the study’s findings (and further extensions) can help improve future conflict data collection. Wars will continue for the foreseeable future, and it is important to understand and quantify their violence as we work to roll them back.

## Abbreviations

IHL: International Humanitarian Law
PHR: Physicians for Human Rights
SAMS: Syrian American Medical Society
